# COTI-2 suppresses the malignancy of bladder cancer by inducing apoptosis via the AMPK-mTOR signaling pathway

**DOI:** 10.22038/ijbms.2024.80284.17378

**Published:** 2025

**Authors:** Yuancai Zheng, Keqi Wang, Chenyu Wu, Yuying Qin, Yihan Sun, Xinyu Lu, Yupeng Xu, Gonghui Li

**Affiliations:** 1 Department of Urology, Sir Run Run Shaw Hospital, School of Medicine, Zhejiang University, Hangzhou, 310000, China; 2 Department of Urology, The First Affiliated Hospital of Wenzhou Medical University, Wenzhou, 325000, China; 3 Department of Pediatrics, The First Affiliated Hospital of Wenzhou Medical University, Wenzhou, 325000, China; 4 The Second Clinical Medical College of Wenzhou Medical University, Wenzhou, 325000, China; 5 The First Clinical Medical College of Wenzhou Medical University, Wenzhou, 325000, China; # These authors contributed equally to this work

**Keywords:** Apoptosis, AMPK/mTOR pathway Bladder cancer, COTI-2, Proliferation

## Abstract

**Objective(s):**

COTI-2, an innovative oral homocysteine, has shown promising antitumor results on multiple types of cancer. However, its effects in treating bladder cancer (BCa) and the underlying molecular mechanisms have not been elucidated. The present study aimed to explore the antitumor effects of COTI-2 on BCa and the potential mechanisms.

**Materials and Methods:**

BCa cell lines, including the 5637 and T24 cell lines, were treated with COTI-2 at concentrations of 0.5 and 1 μM, respectively. Cell Counting Kit (CCK)-8 assay, colony formation assay, apoptosis assay, and transwell migration and invasion assay were conducted to evaluate the antitumor effects of COTI-2 on BCa cells. Western blotting, H&E, immunohistochemical staining, and immunofluorescence analysis were performed to investigate the underlying mechanisms. Moreover, a xenograft model in nude mice using T24 cells was generated to determine the antitumor activities of COTI-2 *in vivo*.

**Results:**

COTI-2 highly inhibited the proliferation of BCa cell lines, including 5637 and T24 cells, and induced their apoptosis. Moreover, it efficiently suppressed the migration and invasion of BCa cells. Additionally, the subcutaneous xenograft model in nude mice showed that COTI-2 treatment inhibited the tumor growth of BCa by inducing its apoptosis in vivo. We also found that COTI-2 promoted apoptosis in BCa cells, presumably through activating the AMPK/mTOR pathway.

**Conclusion:**

Our data suggest that COTI-2 effectively reduces the malignancy of BCa, probably by inducing apoptosis via the AMPK/mTOR signaling pathway. These data highlight the potential of COTI-2 as a therapeutic agent for the treatment of BCa.

## Introduction

Globally, bladder cancer (BCa) remains the most common malignant tumor affecting the urinary system, ranking ninth among all malignant tumors in terms of incidence (1). According to the Global Cancer Research report, 549, 393 individuals were diagnosed with BCa in 2018, with 199, 922 succumbing to this disease (2). The primary treatment for BCa involves surgical resection followed by postoperative adjuvant intravesical instillation (3). However, the recurrence rate for patients with BCa following surgery is alarmingly high, with a 5-year recurrence rate reaching up to 24%-84% (4). Thus, this underscores the critical need to develop new drugs to target and treat BCa effectively.

Apoptosis is a conservative process that plays a crucial role in maintaining the balance between cell survival and death (5). However, abundant apoptotic tumor cells can excessively release damage-associated molecular patterns (DAMPs), cytokines, and chemokines to form an extremely active tumor inflammatory immune microenvironment, resulting in the inhibition of tumor growth and metastasis (6)^.^ Therefore, inducing apoptosis in BCa cells is a good therapeutic strategy for tumor mass and preventing metastasis. 

Several classical signaling pathways, including adenosine monophosphate (AMP) activated protein kinase (AMPK) and mammalian target of rapamycin (mTOR) pathways, are instrumental in regulating the biological process of apoptosis of BCa cells and provide potential therapeutic targets for the treatment of BCa (7, 8). In recent years, AMPK, as a key factor in sensing energy loss and activating apoptosis, has been considered an important molecular target for preventing and treating cancer (9, 10). The activation of AMPK can induce the expression of caspase-3, caspase-8, and caspase-9, control the key processes of cell survival and metabolism, and play a critical role in promoting the apoptosis of BCa cells (11, 12). The mTOR pathway can be activated by growth factors, energy, and other stress signals, and participates in the regulation of cell growth, proliferation, and survival (13, 14). In addition, it has been confirmed that AMPK negatively regulates the activity of mTOR in BCa cells, thus promoting its apoptosis and proliferation inhibition (15, 16). Therefore, regulating the AMPK/mTOR signal pathway might be a promising strategy for treating BCa.

COTI-2 is a novel small-molecule compound believed to regulate the activity of the p53 protein (17, 18). It is undergoing phase I clinical trials for advanced or recurrent gynecological malignancies and head and neck squamous cell carcinoma (HNSCC)(19, 20). Studies have shown that COTI-2 exhibits significant anti-proliferative activities in multiple tumor cell lines and tumor xenograft mouse models as a standalone treatment (21). However, the research concerning the effects of COTI-2 on BCa remains blank. Therefore, further investigation is underway to define its impact on BCa cells and its underlying molecular mechanisms. 

In the present study, we evaluated the effects of COTI-2 on BCa growth inhibition both *in vitro* and *in* *vivo.* We also sought to investigate whether COTI-2 can induce apoptosis by regulating the AMPK-mTOR signaling cascade. By revealing the molecular mechanism by which COTI-2 inhibits the proliferation of human BCa cells, our results may contribute to the development of novel treatments for patients with BCa.

## Materials and Methods

### Cell culture and reagents

Human bladder cancer cell lines (5637 and T24) were obtained from the Shanghai Cell Bank of the Chinese Academy of Sciences. All cells were maintained in DMEM culture medium (Biological Industries, Israel) supplemented with 10% fetal bovine serum (Gemini, USA) in a humidified atmosphere with 5% CO_2_ at 37 ^°^C. The COTI-2 (purity: ≥ 99%, formula: C19H22N6S, CAS No.: 1039455-84-9) was purchased from MCE (Shanghai, China) and dissolved in dimethyl sulfoxide (DMSO, Sigma, St. Louis, MO, USA) at a concentration of 10 mM, and then diluted into culture medium at appropriate concentration depending based on the experiment and the final DMSO concentration was under 0.1%.

### Determination of IC50

The half-maximal inhibitory concentration of COTI-2 in BCa cells was determined by Cell Counting Kit (CCK)-8 (Dojindo, Kumamoto, Japan). BCa cells were inoculated into 96-well plates with 1000 cells per well and cultured overnight. The cells were treated with a culture medium containing different concentrations of COTI-2 for 48 hr. Ten microliter CCK-8 solution was added to each well and incubated for 2 hr. The absorbance was measured at 450 nm by an ELX800 enzyme labeling instrument (Bio-Tek, USA). Finally, the IC_50_ was calculated using GraphPad Prism 8.0 software (GraphPad Software, Inc., La Jolla, CA, USA).

### Cell proliferation

The proliferation ability of BCa cells was measured by a CCK-8 kit (Dojindo, Kumamoto, Japan). In brief, BCa cells were inoculated into 96-well plates with 1000 cells per well and cultured overnight. Then, the cells were treated with a culture medium containing the corresponding concentrations of COTI-2 at 0, 0.5, and 1.0 μM, respectively, and the cell viability was measured at the absorbance of 450 nm at 24, 48, 72, and 96 hr by an ELX800 enzyme labeling instrument (Bio-Tek, USA).

### Cell colony formation

5637 and T24 cells were implanted into 6-well plates (1,000 cells/well) and incubated overnight for adherence. They were then treated with a medium containing COTI-2 at 0, 0.5, and 1.0 μM concentrations, respectively. After the cells formed cell colonies in the incubator for one week, the number of macroscopic colonies was counted by using the Image J software (National Institute of Mental Health, Bethesda, Maryland, USA, version 1.8.0) after cells were fixed with methanol for 20 min and stained with 0.1% crystal violet (Beyotime, Shanghai, China) for 30 min to visualize the colonies.

### Transwell invasion and migration assay

 For the cell invasion assay, 5637 and T24 cells in medium containing 2% fetal bovine serum (Gemini, USA) were inoculated onto a BioCoat Matrigel (8 μm pore size; BD Biosciences, USA, #354234) coated-transwell chambers (Corning, USA, #3422), and the lower chamber was filled with a medium containing 20% fetal bovine serum to induce the invading BCa cells. After 72 hr, the cells that invaded through the insert membrane were fixed with 4% paraformaldehyde for 30 min and stained with 0.1% crystal violet (Beyotime, Shanghai, China) for 15 min. The cell migration assay was conducted using transwell inserts using the same method without supplementing the BioCoat Matrigel. Five random field images were captured from each film under the microscope. The degree of migration and invasion was expressed as the average number of cells in each microscopic field. 

### In vivo xenograft assay

The animal study protocol was reviewed by the Animal Experiments Ethical Committee of Zhejiang University and was approved by the Zhejiang Medical Experimental Animal Care Commission. Six-week-old female BALB/c nude mice were acquired from SLAC Laboratory Animal Co., Ltd. (Shanghai, China) for xenografts. A suspension of T24 cells (5×10^6^ cells) in 0.1 ml of PBS was injected subcutaneously into the lower flank of nude mice. COTI-2 was dissolved in DMSO and then diluted in mineral oil at the final concentration of 10 mg/ml. When the tumors were visible, COTI-2 was intraperitoneally injected at the concentration of 3 mg/kg every other day eight times. The control group was intraperitoneally injected with an equal dose of DMSO in mineral oil. The specific dosage of COTI-2 was determined based on the published literature (20). The mice’s body weight and tumor size were measured every three days. After being monitored for 15 days, the mice were sacrificed, and the tumors were obtained and measured. The tumor volume was calculated by the following formula: volume = (L×W^2^)/2, where ‘L’ and ‘W’ are the longest and shortest diameters of the tumor, respectively. 

### H&E and immunohistochemical staining assays

The harvested tumor specimens were immediately fixed in 4% paraformaldehyde for 48 hr. After dehydration and paraffin embedding, slices of 4.5 μm-thick were processed for hematoxylin-eosin (H&E) staining (G1120, Solarbio, Beijing, China) by standard procedures. For the immunohistochemical staining (IHC) assay, the 4.5μm tumor sections were dewaxed in xylene and rehydrated in graded ethanol. Antigen retrieval was performed in sodium citrate buffer (C8532, Sigma, Missouri, USA), and the endogenous peroxidase activity was inhibited with 3% H2O2. Then the slides were treated with 5% bovine serum albumin (BSA, A1933, Sigma, Missouri, USA) for 30 min, and the sections were incubated at 4 ^°^C overnight with primary antibodies of anti-Ki67 (1:200 dilution; 27309-1-AP, Wuhan, China) and anti-Cleaved C3 (1:200 dilution; 13075-1-AP, Wuhan, China). After washing, the slides were incubated with HRP-conjugated goat anti-rabbit secondary antibody (1:200 dilution; Biosharp, BL003A) for 1 hr at 37 ^°^C and then subjected to a color reaction using 3,3’-diaminobenzidine (DAB) working solution (P0202, Beyotime, Shanghai, China). After counterstaining with hematoxylin for 2 min, the slides were sealed with neutral resin. The images of H&E and IHC staining were photographed under a microscope (DM750, Leica Microsystems, Wetzlar, Germany), and the quantitative analysis of immunostaining results was executed using Image-Pro plus 6.0 software (Media Cybernetics, Bethesda, USA).

### Western blotting

BCa cells were harvested and lysed in radioimmunoprecipitation assay (RIPA) lysis buffer (P0013C, Beyotime, Shanghai, China) containing Protease and Phosphatase Inhibitor Cocktail (ab201119, Abcam, United States) to extract total proteins. The protein concentrations were determined using a bicinchoninic acid (BCA) analysis kit (P0012S, Beyotime, Shanghai, China) according to the manufacturer’s instructions. Then, equal quantities of prepared protein samples were loaded and separated by 10% sodium dodecyl sulfate-polyacrylamide gel electrophoresis (SDS-PAGE) and transferred to a polyvinylidene difluoride (PVDF) membrane (Millipore Corp, USA). Subsequently, the membrane was blocked with 5% skim milk (BD Biosciences) for 2 hr at room temperature and then incubated at 4 ^°^C overnight with the following specific primary antibodies: anti-Cleaved C3 (1:1000 dilution; Abcam, ab2302), anti-BAX (1:1000 dilution; CST, #2772), anti-AMPK (1:1000 dilution; CST, Cat5832s), anti-p-AMPK (1:1000 dilution; Thr172, CST, Cat8208s), anti-mTOR (1:1000 dilution; Abcam, ab87540), anti-p-mTOR (1:1000 dilution; CST, #5536), and anti-GAPDH (1:10,000 dilution; Abcam, ab245355). The membranes were incubated with a horseradish peroxidase-conjugated secondary antibody (1:8000 dilution; Biosharp, BL003A) for 1 hr at room temperature after washing three times with Tris-buffered saline and 0.1% Tween 20 (TBS-T). Finally, the immunodetection was performed with an enhanced chemiluminescence (ECL) solution (WP20005, Thermo Fisher Scientific, California, USA). Protein expression was quantified and analyzed using Image J software (National Institute of Mental Health, Bethesda, Maryland, USA). GAPDH was used as an internal reference.

### Immunofluorescence staining analysis

BCa cells (5,000 cells/well) were seeded in a 12-well chamber slide and grown overnight. After being treated with COTI-2, cells were fixed with 4% paraformaldehyde for 30 min and permeabilized with 0.3% Triton X-100 (Sigma; prepared in PBS) for 15 min at room temperature. Then, cells were blocked with 5% BSA (A1933, Sigma, Missouri, USA) for 1 hr and then incubated with the primary antibodies of anti-Ki67 (1:200 dilution; 27309-1-AP, Wuhan, China) and anti-Cleaved C3 (1:200 dilution; 13075-1-AP, Wuhan, China) at 4 ^°^C overnight. The following day, the cells were washed with PBS and then incubated with goat anti-rabbit secondary antibody coupled with Alexa 488 (1:400 dilution; Abcam, ab150077) at 37 ^°^C for 1 hr. Finally, the nuclei were stained with 4’,6-diamidino-2-phenylindole (DAPI, Beyotime) for 5 min. The resulting images were captured using a confocal microscope (SP8, Leica Microsystems, Wetzlar, Germany).

### Statistical analysis

The data analyzed in this study were normally distributed and presented as the mean±standard deviation (SD). Statistical analyses were done using GraphPad Prism 8.02 software (GraphPad Software, Inc., La Jolla, CA, USA). Students’ unpaired t-test was employed for comparisons between the two groups. One-way analysis of variance (ANOVA) test was used for comparisons between multiple groups, followed by Tukey’s post hoc test. A value of *P*<0.05 was considered statistically significant. All experiments were independently performed at least three times.

## Results

### COTI-2 effectively suppressed cell proliferation of BCa cells in vitro

**Figure 1:**
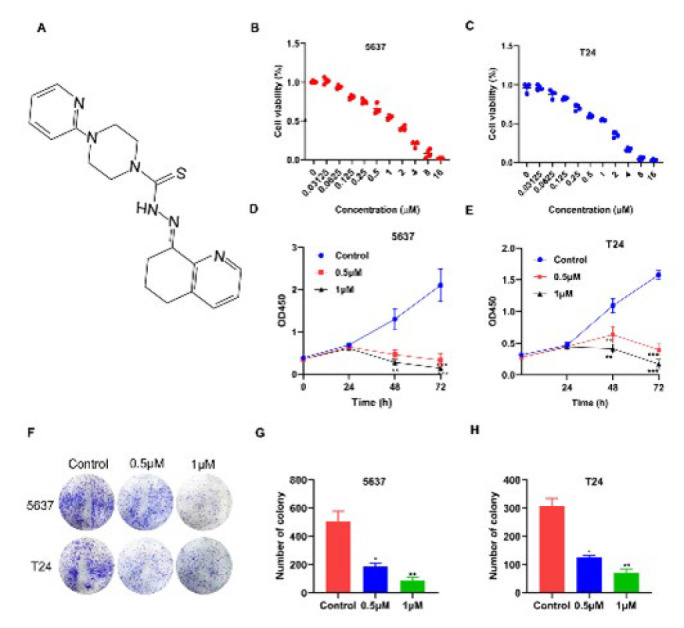
Proliferation inhibition by COTI-2 in 5637 and T24 BCa cells

**Figure 2:**
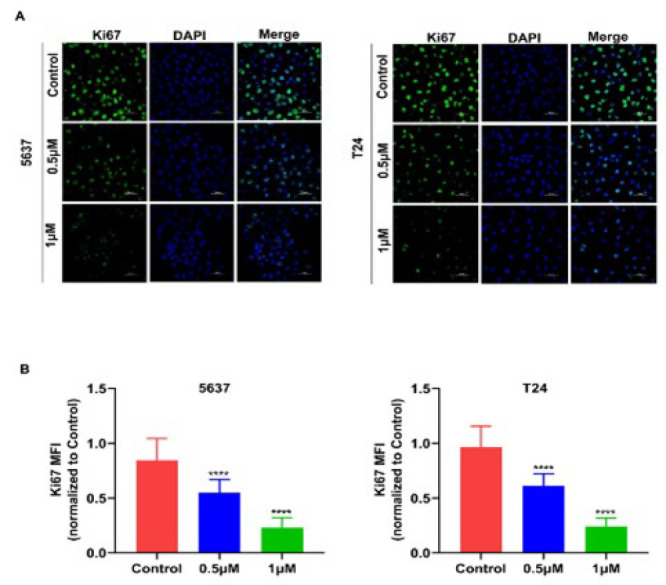
COTI-2 reduces the expression of Ki67 in BCa cells

COTI-2 is a novel oral thiocysteine small molecule compound (17-18), and the chemical structure of COTI-2 is depicted in Figure 1A. To clarify its effects on the growth of human BCa, we first treated BCa cell lines, including the 5637 and T24 cell lines, with different concentrations of COTI-2 for 24 hr. As shown in Figure 1B-1C, the cell survival rate of BCa cells was decreased by COTI-2 treatment in a dose-dependent manner. In addition, the IC50 value of COTI-2 on 5637 and T24 BCa cells for 24 hr was found to be 0.526 and 0.532 μM, respectively (Figure 1B-1C). Therefore, concentrations of 0.5 and 1 μM of COTI-2 were selected for further study. The CCK8 assay showed that treatment with COTI-2 (0.5 and 1 μM) resulted in a time-dependent growth suppression in 5637 and T24 cells (Figure 1D and 1E). The colony formation assay also demonstrated that COTI-2-treated cells had fewer colonies than the control cells (Figure 1F-1H). Furthermore, the effects of COTI-2 on the expression of Ki67 (a marker of cell proliferation) in BCa cells were also investigated. Immunofluorescence analysis revealed a significant reduction in the green fluorescence intensity of Ki67 in the COTI-2-treated group compared to the untreated group (Figure 2A-2B), which was consistent with the results of CCK8 detection. Collectively, the above data suggest that COTI-2 suppresses the proliferation of human BCa cells *in vitro. *

### COTI-2 induces apoptosis in BCa cells

**Figure 3:**
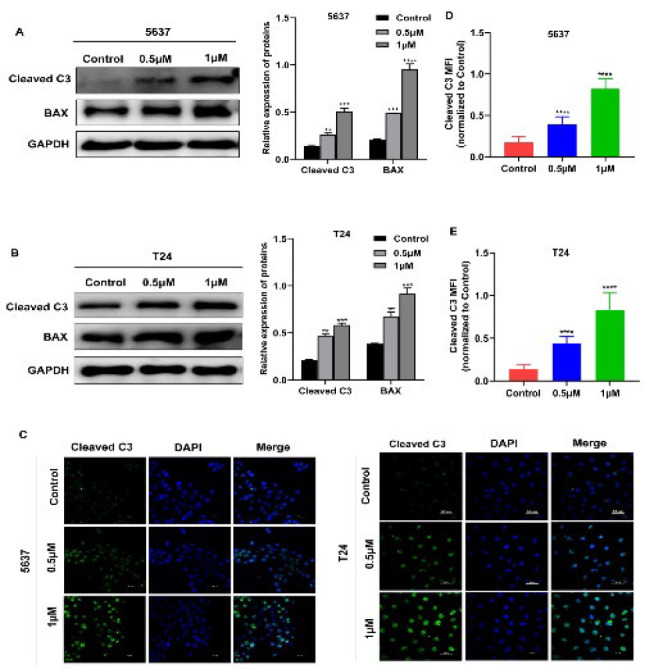
COTI-2 induces apoptosis of BCa cells

Next, we investigated the pro-apoptotic capability of COTI-2 on BCa cells. Thus, the protein expression levels of pro-apoptotic indicators Cleaved C3 and Bax were measured by Western blotting in BCa cells. As shown in Figure 3A-3B, the expressions of these two pro-apoptotic markers were significantly increased in 5637 and T24 cells treated with COTI-2. In addition, immunofluorescence staining revealed an obvious elevation in the green fluorescence intensity of Cleaved C3 in the COTI-2-treated group compared to the untreated group (Figure 3C-3E). These data suggest that COTI-2 supplementation reveals a promotional role in BCa cell apoptosis.

### COTI-2 inhibits the migration and invasion rate of BCa cells in vitro

**Figure 4:**
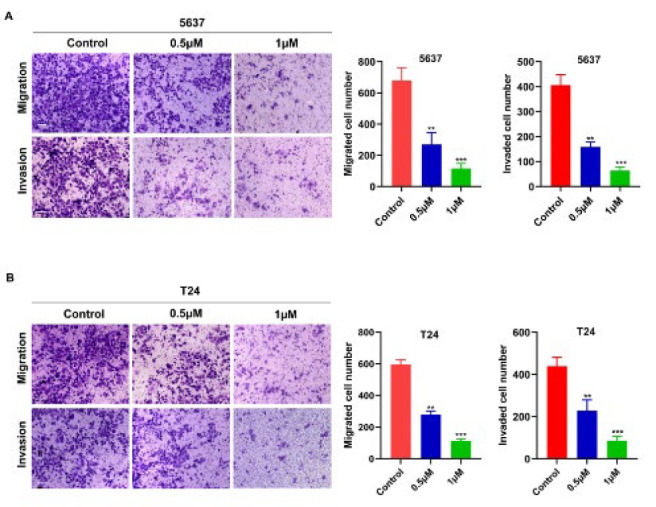
COTI-2 weakens the migration and invasive ability of BCa cells^.^ (A) 5637 cells were treated with 0, 0.5, and 1.0 μm COTI-2 for 48 hr for transwell migration assay and 72 hr for transwell invasion assay (scale bar=100 μm). (B) T24 cells were treated with 0, 0.5, and 1.0 μm COTI-2 for 48 hr for transwell migration assay and 72 hr for transwell invasion assay (scale bar=100 μm). Quantitative data were expressed as the mean±SD; **P<0.01, ***P<0.001 vs the control group

Given that apoptosis of tumor cells contributes to the inhibition of cancer metastasis (5-6), we explored whether COTI-2 had an inhibitory effect on the migration and invasion capacities of BCa cells. Transwell chambers with or without supplement of the BioCoat Matrigel were used to investigate the effects of COTI-2 on BCa invasion and migration. As shown in Figures 4A and 4B, treatment with COTI-2 resulted in a significant reduction in cell migratory and invasive capacities in 5637 and T24 cells compared to those in parental cells, respectively. These results demonstrate that COTI-2 effectively reduces the malignant characteristics of BCa cells.

### COTI-2 regulates the AMPK/mTOR signaling pathway in BCa cells

**Figure 5:**
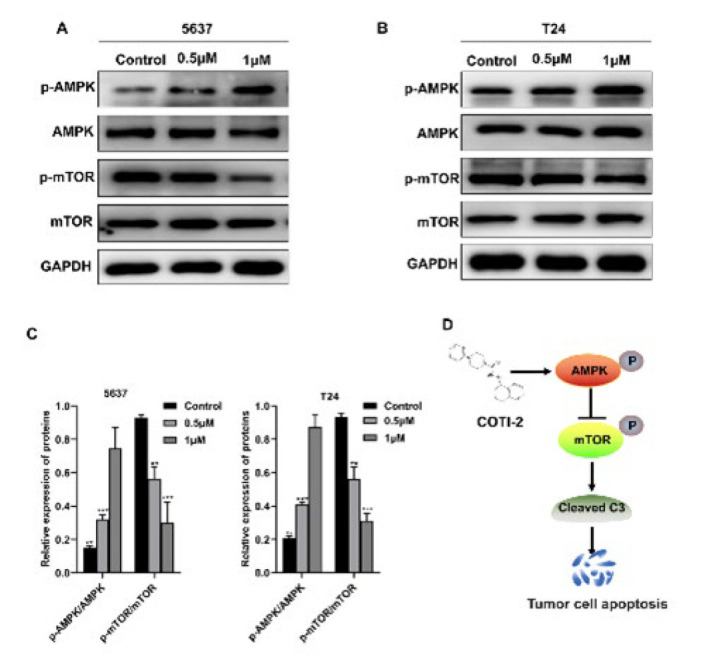
COTI-2 activates the AMPK/mTOR signaling pathway in BCa cells

Previous studies strongly demonstrate that the AMPK/mTOR signaling pathway plays a key role in regulating apoptosis and proliferation of BCa cells (15-16). In this study, we sought to determine whether COTI-2 regulates the AMPK/mTOR signaling pathway in BCa cells. Western blot analysis showed that COTI-2 treatment in 5637 and T24 BCa cell lines led to a remarkable dose-dependent up-regulation in the levels of AMPK phosphorylation (indicating the activation of AMPK). Moreover, a dose-dependent reduction of mTOR phosphorylation (indicating the activation of mTOR) levels was observed in these two BCa cell lines after being treated with COTI-2 (Figures 5A-5C). These data indicate that COTI-2 probably exerts its antitumor effects in BCa cells by regulating the AMPK/mTOR signaling pathway.

### COTI-2 inhibits tumor growth of BCa in vivo

**Figure 6:**
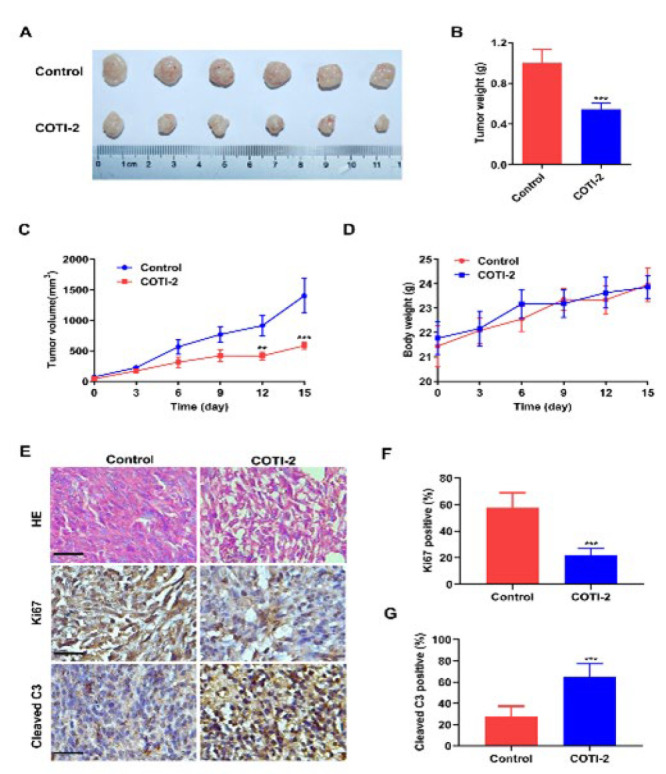
COTI-2 inhibits the growth of T24 xenografts in nude mice

To determine whether COTI-2 has a similar inhibitory effect on the growth of BCa *in vivo*, we created a xenograft model in nude mice. This involved injecting COTI-2 intraperitoneally at a concentration of 3 mg/kg or an equal volume of vehicle into the mice every other day for a total of eight injections. As shown in Figure 6A-6C, the tumor volumes and weights were significantly reduced under COTI-2 treatment. Additionally, all mice tolerated the supplementation of COTI-2 well without body weight loss (Figure 6D). H&E staining presented a lower density of tumor cells and a higher density of necrotic cells after COTI-2 treatment in the xenograft tumor sections compared to the control group (Figure 6E). Immunohistochemistry assay of the xenograft tumor sections indicated that COTI-2 treatment significantly decreased the expression of Ki-67 and increased the expression of Cleaved C3 (Figure 6E-6G). Thus, these data suggest that BCa treated with COTI-2 significantly reduced tumor growth and apoptosis induction *in vivo*.

## Discussion

In clinic, radical cystectomy is still the only treatment option after the recurrence of BCa, which will greatly reduce the quality of life in patients (22, 23). Therefore, there is an urgent need to identify new drug targets and find an effective and safe drug to treat BCa better. 

COTI-2, a novel small-molecule anticancer drug identified by the proprietary computing platform called CHEMSAS, has been proven to have obvious antitumor effects on cancers such as lung cancer, triple-negative breast cancer, and HNSCC (20, 21, 24). Low dose of COTI-2 can significantly inhibit the proliferation of lung cancer cell line SHP-77, mainly inducing apoptosis and necrosis of most cells by reducing the levels of p-AKT (21). Previous studies have also reported that COTI-2 can target and reactivate mutant p53 and inhibit the growth of breast cancer cells in triple-negative breast cancer (24, 25). In addition, COTI-2 induces DNA damage and leads to cell apoptosis by p53-dependent and independent mechanisms* in vitro* in HNSCC cells (20). However, the tumor-killing effects of COTI-2 in BCa have not been confirmed. In this research, we investigated the effects of COTI-2 on the growth of 5637 and T24 cells by CCK8 and colony formation assay. Our findings exhibited that COTI-2 considerably inhibits the proliferation of these two cell types. In addition, the immunofluorescence results showed that the expression of proliferation-related protein Ki67 in COTI-2-treated cells was significantly lower than that in the control group. Moreover, we also found that COTI-2 had inhibitory effects on the migration and invasion capacities of BCa cells. Our data demonstrated that COTI-2 has impressing inhibitory roles in the proliferation, migration, and invasion of BCa cells.

Next, we explored the molecular mechanism by which COTI-2 confers the antitumor effects in BCa cells. Among the many potential mechanisms responsible for inhibiting tumor growth and metastasis, the pro-apoptosis mechanism is considered a central factor for restraining proliferation, migration, and invasion of BCa cells (26-28). Thus, we investigated whether COTI-2 regulates apoptosis in BCa cells. Unsurprisingly, the apoptosis-inducing effect of COTI-2 on 5637 and T24 BCa cell lines was confirmed by Western blotting and immunofluorescence staining, as evidenced by significantly up-regulated expression of pro-apoptotic proteins (Cleaved C3 and Bax) in both BCa cell lines, indicating that COTI-2 plays a promotional role in BCa cell apoptosis. Therefore, we infer that the antitumor effects of COTI-2 in BCa cells are associated with its pro-apoptosis actions.

Among the intracellular signaling systems that regulate the balance between proliferation and apoptosis of BCa cells, the AMPK/mTOR signaling pathway is particularly important (28, 29). Compared with normal tissue cells, tumor cells grow faster with exuberant energy metabolism (10). AMPK is an important regulator of energy balance in tumor cells (9, 10, 30). The activation of AMPK and its downstream signal cascade coordinate the dynamic changes of tumor cell bioenergy, exhibiting a pivotal role in tumor development and progression by regulating metabolic pathways (31, 32). Additionally, mTOR is one of the downstream targets of AMPK as an intracellular nutrition sensor to control protein synthesis, cell death, and metabolic function (33). When the tumor cells are deficient in nutrients, the intracellular energy is insufficient, ATP decreases, and AMPK production increases, resulting in the activation of AMPK and the inhibition of mTOR, thus inducing the expression and activation of Caspase-3, of which activation is considered to be the last and irreversible step in the process of apoptosis (34), leading to apoptosis and proliferation inhibition of tumor cells (31, 35). In the current study, COTI-2 supplementation activated the AMPK/mTOR signaling pathway in 5637 and T24 cells in a dose-dependent manner, as indicated by elevated levels of AMPK phosphorylation and decreased levels of mTOR phosphorylation, respectively. These findings suggest that the antitumor actions of COTI-2 in BCa cells were probably through the AMPK/mTOR signaling pathway.

Drug-induced toxicity is a major limiting factor for the success of all cancer treatments, including cytotoxic chemotherapy, targeted therapy, and, more recently, immunotherapy (36, 37). In a xenograft model of SHP-77 small cell lung cancer cell line, COTI-2 could significantly inhibit tumor growth at a dose as low as 3 mg/kg. In contrast, the dose did not significantly affect body weight loss or any obvious disease signs in nude mice (21). Moreover, certain studies have shown that COTI-2 has a better inhibitory effect on the proliferation of colorectal cancer cells than cetuximab and erlotinib, and COTI-2 is more effective than cisplatin and paclitaxel in the treatment of small cell lung cancer (21). The above evidence suggests that COTI-2 may have better safety and efficacy in treating cancer than other traditional chemotherapy drugs. Similarly, in our established BCa xenograft model, the growth trend of tumor volume in COTI-2 treated mice was significantly slower than that in the control group at a dose of 3 mg/kg without body weight loss. In addition, COTI-2-treated mice showed significantly decreased expression of Ki-67 and increased expression of Cleaved C3 in the xenograft tumor sections, indicating that COTI-2 could inhibit tumor proliferation and induce apoptosis of BCa *in vivo*. These data suggest that COTI-2 may support a promising drug for treating BCa.

(A) Chemical structure of COTI-2. (B and C) Cell viability after incubation with COTI-2 (0, 0.3125, 0.0625, 0.125, 0.25, 0.5, 1, 2, 4, 8, and 16 μM) for 24 hr in 5637 and T24 cells. (D and E) Effect of COTI-2 (0.5 and 1μM) on the proliferation of 5637 and T24 cells was detected by CCK-8 assay. (F-H) 5637 and T24 cells were inoculated in six-well plates and treated with 0, 0.5, and 1.0 μm COTI-2, respectively, and then, colony formation assay was performed by crystal violet staining after seven days of culture, and the histogram is the statistical number of colonies. Quantitative data were expressed as the mean±SD; *P<0.05, **P<0.01, ***P<0.001 vs the control group

(A) After treating 5637 and T24 cells with 0, 0.5, and 1.0 μm COTI-2 for 24 hr, the immunofluorescence assay of Ki67 showed cell proliferation at different COTI-2 concentrations (scale bar=50 μm). (B) Quantitative analysis of Ki67 immunofluorescence was performed by Image J software. Quantitative data were expressed as the mean±SD; ****P<0.0001 vs the control group

(A-B) Western blotting analysis displayed the expression changes of Cleaved C3 and BAX in 5637 and T24 cells, and densitometric quantification was performed using Image J. GAPDH was used as the loading control. (C-E) After respectively treating 5637 and T24 cells with 0, 0.5, and 1.0 μm COTI-2 for 24 hr, the immunofluorescence assay of Cleaved C3 showed cell apoptosis at different COTI-2 concentrations (scale bar=50 μm) and the quantitative analysis of Cleaved C3 immunofluorescence was performed using Image J software. Quantitative data were expressed as the mean±SD; **P<0.01, ***P<0.001, ****P<0.0001 vs the control group

(A-C) Western blotting analysis displayed the expression changes of vital molecules in the AMPK/mTOR signaling pathway in 5637 and T24 cells, and densitometric quantification was performed using Image J. GAPDH was used as the loading control. (D) Mechanistic illustration of the antitumor effects of COTI-2 in BCa cells. Quantitative data were expressed as the mean±SD; **P<0.01, ***P<0.001 vs the control group

(A) Establishment of the subcutaneous xenograft model through the hypodermic injection with T24 cells and the representative macroscopic view of dissected tumors was presented. (B and C) Dissected tumor weights and volume were evaluated. (D) Body weight of the nude mice was monitored every 3 days after the indicated treatments. (E) H&E staining was performed to assess the histological structure of xenograft tumor sections (scale bar=50 μm), and the expression levels of Ki-67 and Cleaved C3 in tumor tissues were detected by IHC assay (scale bar=50 μm). (F-G) Quantitative analysis of Ki67 and Cleaved C3 in the xenograft tumor sections was performed using Image J software. Quantitative data were expressed as the mean±SD; **P<0.01, ***P<0.001 vs the control group

## Conclusion

In summary, our findings confirm the novel function of COTI-2 in inhibiting the proliferation of BCa cells *in vitro* and *in vivo.* Importantly, this study broadens our understanding of how COTI-2 exerts its antitumor effects in BCa. COTI-2 induces AMPK activation, which in turn promotes the inhibition of mTOR activity, thereby inducing the expression and activation of Caspase-3 and leading to apoptosis of BCa cells (Figures 5D), and thus, is crucial for inhibiting the proliferation, migration, and invasion of BCa cells. In conclusion, because COTI-2 is already in clinical trials for gynecological malignancies and HNSCC and showed better safety and stronger antitumor activity compared with some first-line chemotherapeutic drugs and targeted therapeutic drugs (19, 20), suggesting it has high potential as a novel therapeutic drug in the treatment of BCa.

## Data Availability

All data and materials approved for the present study’s results are available from the corresponding author upon reasonable request.

## Declaration

We have not used any AI tools or technologies to prepare this manuscript.

## References

[1] Antoni S, Ferlay J, Soerjomataram I, Znaor A, Jemal A, Bray F (2017). Bladder cancer incidence and mortality: A global overview and recent trends. Eur Urol.

[2] Bray F, Ferlay J, Soerjomataram I, Siegel RL, Torre LA, Jemal A (2018). Global cancer statistics 2018: Globocan estimates of incidence and mortality worldwide for 36 cancers in 185 countries. CA Cancer J Clin.

[3] Gerber DE (2008). Targeted therapies: A new generation of cancer treatments. Am Fam Physician.

[4] Comperat E, Amin MB, Cathomas R, Choudhury A, De Santis M, Kamat A (2022). Current best practice for bladder cancer: A narrative review of diagnostics and treatments. Lancet.

[5] Morana O, Wood W, Gregory CD (2022). The apoptosis paradox in cancer. Int J Mol Sci.

[6] Gregory CD, Ford CA, Voss JJ (2016). Microenvironmental effects of cell death in malignant disease. Adv Exp Med Biol.

[7] Wang F, Wu H, Fan M, Yu R, Zhang Y, Liu J (2020). Sodium butyrate inhibits migration and induces ampk-mtor pathway-dependent autophagy and ros-mediated apoptosis via the mir-139-5p/bmi-1 axis in human bladder cancer cells. FASEB J.

[8] Sun Y, Berleth N, Wu W, Schlutermann D, Deitersen J, Stuhldreier F (2021). Fin56-induced ferroptosis is supported by autophagy-mediated gpx4 degradation and functions synergistically with mtor inhibition to kill bladder cancer cells. Cell Death Dis.

[9] Keerthana CK, Rayginia TP, Shifana SC, Anto NP, Kalimuthu K, Isakov N (2023). The role of ampk in cancer metabolism and its impact on the immunomodulation of the tumor microenvironment. Front Immunol.

[10] Luo Z, Zang M, Guo W (2010). Ampk as a metabolic tumor suppressor: Control of metabolism and cell growth. Future Oncol.

[11] Gao D, Wang R, Gong Y, Yu X, Niu Q, Yang E (2023). Cab39 promotes cisplatin resistance in bladder cancer via the lkb1-ampk-lc3 pathway. Free Radic Biol Med.

[12] Huang Y, Zhou S, He C, Deng J, Tao T, Su Q (2018). Phenformin alone or combined with gefitinib inhibits bladder cancer via ampk and egfr pathways. Cancer Commun (Lond).

[13] Kim LC, Cook RS, Chen J (2017). Mtorc1 and mtorc2 in cancer and the tumor microenvironment. Oncogene.

[14] Populo H, Lopes JM, Soares P (2012). The mtor signalling pathway in human cancer. Int J Mol Sci.

[15] Wang F, Cao M, Fan M, Wu H, Huang W, Zhang Y (2020). Ampk-mtor-ulk1 axis activation-dependent autophagy promotes hydroxycamptothecin-induced apoptosis in human bladder cancer cells. J Cell Physiol.

[16] Zhang Y, He N, Zhou X, Wang F, Cai H, Huang SH (2021). Betulinic acid induces autophagy-dependent apoptosis via bmi-1/ros/ampk-mtor-ulk1 axis in human bladder cancer cells. Aging (Albany NY).

[17] Nagourney AJ, Gipoor JB, Evans SS, D’Amora P, Duesberg MS, Bernard PJ (2023). Therapeutic targeting of p53: A comparative analysis of apr-246 and coti-2 in human tumor primary culture 3-d explants. Genes (Basel).

[18] Duffy MJ, Synnott NC, Crown J (2018). Mutant p53 in breast cancer: Potential as a therapeutic target and biomarker. Breast Cancer Res Treat.

[19] Westin Shannon N, Wilberto Nieves-Neira Christian Lynam, Salim Kowthar Y, Silva Alison D, Ho Richard T (2018). Safety and early efficacy signals for COTI-2, an orally available small molecule targeting p53, in a phase I trial of recurrent gynecologic cancer. Cancer Res.

[20] Lindemann A, Patel AA, Silver NL, Tang L, Liu Z, Wang L (2019). Coti-2, a novel thiosemicarbazone derivative, exhibits antitumor activity in hnscc through p53-dependent and -independent mechanisms. Clin Cancer Res.

[21] Salim KY, Maleki Vareki S, Danter WR, Koropatnick J (2016). Coti-2, a novel small molecule that is active against multiple human cancer cell lines in vitro and in vivo. Oncotarget.

[22] Al Hussein Al Awamlh B, Chang SS (2023). Novel therapies for high-risk non-muscle invasive bladder cancer. Curr Oncol Rep.

[23] Martinez Rodriguez RH, Buisan Rueda O, Ibarz L (2017). Bladder cancer: Present and future. Med Clin (Barc).

[24] Synnott NC, O’Connell D, Crown J, Duffy MJ (2020). Coti-2 reactivates mutant p53 and inhibits growth of triple-negative breast cancer cells. Breast Cancer Res Treat.

[25] Tang M, Crown J, Duffy MJ (2023). Degradation of myc by the mutant p53 reactivator drug, coti-2 in breast cancer cells. Invest New Drugs.

[26] Perabo FG, Lindner H, Schmidt D, Huebner D, Blatter J, Fimmers R (2003). Preclinical evaluation of gemcitabine/paclitaxel-interactions in human bladder cancer lines. Anticancer Res.

[27] McKnight JJ, Gray SB, O’Kane HF, Johnston SR, Williamson KE (2005). Apoptosis and chemotherapy for bladder cancer. J Urol.

[28] Wu P, Liu S, Su J, Chen J, Li L, Zhang R (2017). Apoptosis triggered by isoquercitrin in bladder cancer cells by activating the ampk-activated protein kinase pathway. Food Funct.

[29] Zhou X, Chen Y, Wang F, Wu H, Zhang Y, Liu J (2020). Artesunate induces autophagy dependent apoptosis through up-regulating ros and activating ampk-mtor-ulk1 axis in human bladder cancer cells. Chem Biol Interact.

[30] Villanueva-Paz M, Cotan D, Garrido-Maraver J, Oropesa-Avila M, de la Mata M, Delgado-Pavon A (2016). Ampk regulation of cell growth, apoptosis, autophagy, and bioenergetics. Exp Suppl.

[31] Penugurti V, Mishra YG, Manavathi B (2022). Ampk: An odyssey of a metabolic regulator, a tumor suppressor, and now a contextual oncogene. Biochim Biophys Acta Rev Cancer.

[32] Trefts E, Shaw RJ (2021). Ampk: Restoring metabolic homeostasis over space and time. Mol Cell.

[33] Visnjic D, Dembitz V, Lalic H (2019). The role of ampk/mtor modulators in the therapy of acute myeloid leukemia. Curr Med Chem.

[34] Ke H, Wang X, Zhou Z, Ai W, Wu Z, Zhang Y (2021). Effect of weimaining on apoptosis and caspase-3 expression in a breast cancer mouse model. J Ethnopharmacol.

[35] Xie Y, Lei X, Zhao G, Guo R, Cui N (2023). Mtor in programmed cell death and its therapeutic implications. Cytokine Growth Factor Rev.

[36] Deutsch A, Leboeuf NR, Lacouture ME, McLellan BN (2020). Dermatologic adverse events of systemic anticancer therapies: Cytotoxic chemotherapy, targeted therapy, and immunotherapy. Am Soc Clin Oncol Educ Book.

[37] Zhang Y, Li Y, Fu Q, Han Z, Wang D, Umar Shinge SA (2023). Combined immunotherapy and targeted therapies for cancer treatment: Recent advances and future perspectives. Curr Cancer Drug Targets.

